# Efficacy and safety of glecaprevir/pibrentasvir in Japanese patients with chronic genotype 1 hepatitis C virus infection with and without cirrhosis

**DOI:** 10.1007/s00535-017-1391-5

**Published:** 2017-09-25

**Authors:** Kazuaki Chayama, Fumitaka Suzuki, Yoshiyasu Karino, Yoshiiku Kawakami, Ken Sato, Tomofumi Atarashi, Atsushi Naganuma, Tsunamasa Watanabe, Yuichiro Eguchi, Hitoshi Yoshiji, Masataka Seike, Yoshiyuki Takei, Koji Kato, Katia Alves, Margaret Burroughs, Rebecca Redman, David L. Pugatch, Tami J. Pilot-Matias, Preethi Krishnan, Rajneet K. Oberoi, Wangang Xie, Hiromitsu Kumada

**Affiliations:** 1 Hiroshima University Hospital, Hiroshima, Japan; 2 Toranomon Hospital, Tokyo, Japan; 3 JA Hokkaido P.W.F.A.C Sapporo-Kosei General Hospital, Sapporo, Japan; 4 Gunma University Hospital, Maebashi, Japan; 5 JA Hokkaido P.W.F.A.C Obihiro-Kosei General Hospital, Obihiro, Japan; 6 NHO Takasaki General Medical Center, Takasaki, Japan; 7 St. Marianna University School of Medicine, Kawasaki, Japan; 8 Saga University Hospital, Saga, Japan; 9 Nara Medical University Hospital, Kashihara, Japan; 10 Oita University Hospital, Yufu, Japan; 11 Mie University Hospital, Tsu, Japan; 12 AbbVie Inc, North Chicago, IL USA

**Keywords:** Hepatitis C virus, Direct-acting antivirals, Glecaprevir/pibrentasvir

## Abstract

**Background:**

The once-daily, all oral, RBV-free, pangenotypic direct-acting anti-viral regimen consisting of co-formulated NS3/4A protease inhibitor glecaprevir and NS5A inhibitor pibrentasvir (G/P), demonstrated high rates of sustained virologic response (SVR) in phase 2 and 3 studies outside Japan.

**Methods:**

CERTAIN-1 is a phase 3, open-label, multicenter study assessing the safety and efficacy of G/P (300/120 mg) once daily in Japanese patients with chronic HCV GT1 infection. Patients without cirrhosis received 8 weeks of G/P or 12 weeks of ombitasvir/paritaprevir/ritonavir (OBV/PTV/r, 25/150/100 mg); patients with cirrhosis received G/P for 12 weeks. The primary efficacy endpoint was non-inferiority of G/P compared to OBV/PTV/r by assessing SVR at post-treatment week 12 (SVR12) among non-cirrhotic patients without the NS5A Y93H polymorphism.

**Results:**

SVR12 was achieved by 128/129 (99.2%; one patient lost to follow-up) non-cirrhotic patients in the 8-week G/P Arm (including 23/23 patients with the NS5A Y93H polymorphism) and 52/52 (100%) patients in the 12-week OBV/PTV/r Arm. No patients from the G/P Arm prematurely discontinued the study drug or experienced a treatment-emergent serious adverse event (TESAE). Three patients from the OBV/PTV/r Arm experienced five TESAEs and one of these patients discontinued the study drug due to TESAEs. All 38 (100%) patients with compensated cirrhosis achieved SVR12; in this group, no TESAEs were reported and one patient discontinued treatment due to an AE.

**Conclusions:**

CERTAIN-1 study results demonstrate high efficacy and favorable tolerability of G/P in GT1-infected Japanese patients including those with the NS5A Y93H polymorphism, with no virologic failures observed.

**Electronic supplementary material:**

The online version of this article (doi:10.1007/s00535-017-1391-5) contains supplementary material, which is available to authorized users.

## Introduction

Approximately 1.5 million individuals are estimated to have HCV infection in Japan, which corresponds to the highest rate in the industrialized world [1–4]. Of those, approximately 70% are infected with GT1 (predominantly GT1b) and 30% with GT2 [5]. When left untreated, HCV infection can lead to liver cirrhosis, hepatocellular carcinoma (HCC), and end-stage liver disease [6, 7]. The health burden of chronic HCV infection in Japan is expected to rise over the next several years due to disease progression combined with an aging population [8].

Combinations of direct-acting antiviral agents (DAAs) targeting multiple components of the HCV viral replication process, including NS3/4A, NS5A, and NS5B, have demonstrated high rates of sustained virologic response (SVR) in Japanese patients with chronic HCV GT1 infection. The 2-DAA regimen ombitasvir (OBV)/paritaprevir (PTV)/ritonavir (r) (ritonavir is a pharmacoenhancer with no anti-HCV activity; PTV identified by AbbVie and Enanta) administered once daily for 12 weeks, achieved SVR rates of 95–98% in GT1b HCV-infected Japanese patients without cirrhosis and 91% in patients with compensated cirrhosis [9]. The Japanese Society of Hepatology (JSH) currently recommends OBV/PTV/r, sofosbuvir (SOF)/ledipasvir (LDV), grazoprevir (GZR)+elbasvir (EBR), or daclatasvir (DCV)/asunaprevir (ASV)/beclabuvir (BCV) administered for 12 weeks [10, 11] as first-line treatment regimens for interferon/pegylated interferon (IFN/pegIFN)+ribavirin (RBV) treatment-naïve and treatment-experienced patients, including those with compensated cirrhosis [12–14]. Despite these available regimens to treat patients with GT1 infection, shorter treatment durations can result in less adverse events and better compliance [15]. Furthermore, SOF/LDV is not recommended for use in patients with severe renal impairment [14], OBV/PTV/r is not recommended for use in patients with baseline polymorphisms in NS5A at position Y93 [13], while DCV/ASV/BCV requires weekly liver function monitoring [11].

Glecaprevir (GLE, formerly ABT-493, identified by AbbVie and Enanta), an NS3/4A protease inhibitor co-formulated with pibrentasvir (PIB, formerly ABT-530), an NS5A inhibitor, is currently being evaluated as a pangenotypic regimen (G/P). Preclinical and previous clinical studies have demonstrated that this combination regimen has a high barrier to resistance and potency against common NS3 and NS5A polymorphisms, including L31 M/V and Y93H in NS5A [16]. High efficacy of G/P in various patient populations over short treatment durations compared to currently recommended treatments have been demonstrated outside Japan, including in patients who have been previously considered difficult to treat. Here we describe the safety and efficacy of G/P administered for 8 weeks compared with OBV/PTV/r administered for 12 weeks in GT1 HCV-infected Japanese patients without cirrhosis, and a separate cohort of patients with compensated cirrhosis who received G/P for 12 weeks.

## Methods

### Study design

**Fig. 1:**
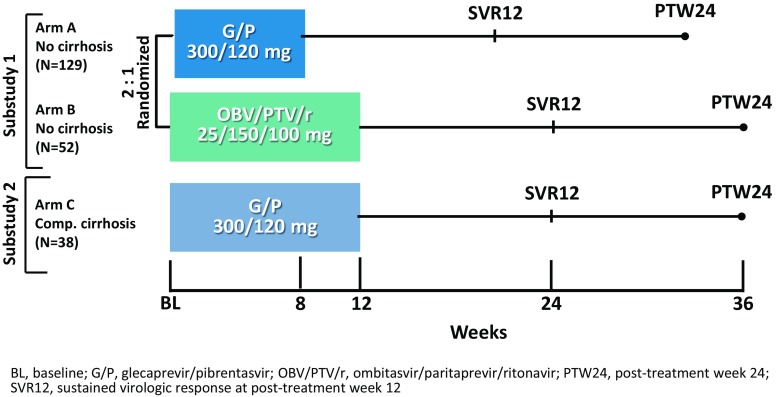
Study design for GT1-infected DAA-naïve patients enrolled and treated in the CERTAIN-1 study (Arm C included other patient cohorts reported elsewhere)

CERTAIN-1 (NCT02707952) is a phase 3, open-label, multicenter study assessing the safety and efficacy of G/P (300/120 mg) once daily in Japanese patients with HCV infection. The study was composed of two substudies; in Substudy 1, DAA-naïve patients with GT1 HCV infection without cirrhosis and without the Y93H polymorphism were randomized to 8 weeks of treatment with G/P (Arm A) or 12 weeks of treatment with OBV/PTV/r (Arm B). The randomization was stratified by prior IFN-experience (naïve versus experienced) and HCV RNA viral load (< or ≥ 6 million IU/ml). Patients without the NS5A Y93H polymorphism were randomized 2:1 to Arms A or B, while patients with the Y93H polymorphism were assigned to Arm A only. In Substudy 2, DAA-naïve patients with GT1 HCV infection with compensated cirrhosis were assigned to 12 weeks of G/P (300/120 mg) once daily. All patients reported here were required to have an eGFR ≥ 30 ml/min/1.73 m^2^. Substudy 2 also enrolled cohorts of patients other than those with GT1 infection and cirrhosis, including those with eGFR < 30 m/min/1.73 m^2^ and results will be reported elsewhere. All patients were followed for 24 weeks after the last dose of study drug. Figure 1 shows the study design. The treatment duration of G/P for patients without cirrhosis and with compensated cirrhosis was based on both the results from the global Phase 2 SURVEYOR-I and SURVEYOR-II studies [17, 18], and the clinical exposure-response simulation predicted range (data not shown).

All patients provided written, informed consent to participate and the study was conducted consistent with the ethical guidelines of the Declaration of Helsinki and the International Conference on Harmonisation Good Clinical Practice Guidelines. The study was approved by an institutional review board of each study site prior to the initiation of any screening or study-specific procedures. Supporting Fig. 1 shows patient disposition.

### Patients

Patients were screened from February 22, 2016 to June 1, 2016 at 62 study sites in Japan. Adults ≥ 18 years of age with GT1 HCV infection without cirrhosis were eligible for enrollment in CERTAIN-1 Substudy 1 and those with GT1 HCV infection and compensated cirrhosis were eligible for enrollment in CERTAIN-1 Substudy 2. Patients were required to be either HCV treatment-naïve or to have failed prior IFN/pegIFN ± RBV therapy, be DAA-naïve, test positive for anti-HCV Ab, have a plasma HCV RNA load ≥ 1000 IU/ml at the time of screening and have a laboratory result indicating HCV infection with GT1 only. HCV NS5A population sequencing was performed by a central laboratory at the screening visit for Substudy 1 patients to detect the presence or absence of the Y93H polymorphism (approximately 15% detection threshold for Y93H).

In Substudy 1, patients were required to demonstrate absence of cirrhosis with one of the following criteria: a liver biopsy demonstrating absence of cirrhosis (e.g., Metavir score of ≤ 3 or an Ishak score of ≤ 4), a Fibroscan score < 12.5 kPa, or FibroTest score ≤ 0.72 and aspartate aminotransferase to Platelet Ratio Index ≤ 2. Patients with compensated cirrhosis enrolled in Substudy 2 were required to have one of the following: a liver biopsy with a METAVIR (or equivalent) fibrosis score > 3 or Ishak fibrosis score > 4, a FibroTest score ≥ 0.73 with an aminotransferase to Platelet Ratio Index > 2, a FibroScan score ≥ 14.6 kPa or screening Discriminant Score (z) greater than zero. Absence of HCC was confirmed with a negative ultrasound, computed tomography scan or magnetic resonance imaging scan within 3 months prior to screening or a negative ultrasound at screening. HCV genotype was assessed with the Versant^®^ HCV Genotype Inno LiPA Assay, version 2.0 or higher; if the assay failed to yield a result, a Sanger sequencing assay of the NS5B region was used.

Patients were excluded from this study if they had a positive test result for hepatitis B surface antigen, or anti-human immunodeficiency virus antibody, any current or past clinical evidence of Child–Pugh B or C classification or clinical history of decompensated liver disease such as ascites, hepatic encephalopathy or variceal bleeding, any cause of liver disease other than HCV infection, any clinically significant abnormalities or co-morbidities that make the patient an unsuitable candidate for this study in the opinion of the investigator, or abnormal screening laboratory results as listed in Table 1.

**Table 1:** Abnormal laboratory results exclusion criteria for patients without cirrhosis and with compensated cirrhosis

Assessment	No cirrhosis	Compensated cirrhosis
eGFR^a^, ml/min/1.73 m^2^	< 30	< 30
Serum albumin, g/dL	< LLN	< 2.8
INR	≥ 1.2	≥ 1.8
Hemoglobin, g/dL	< 10	< 10
Platelet count, cells/mm^3^	< 90,000	< 50,000

*INR* international normalized ratio, *LLN* lower limit of normal

^a^ eGFR, estimated glomerular filtration rate (using the MDRD method modified for Japanese population: eGFR = 194 × serum creatinine^−1.094^ × Age^−0.287^ × 0.739 [if female])

### Study assessments

Virologic response was assessed using serum HCV RNA concentration with a lower limit of quantitation of 15 IU/ml. Samples were collected at the screening visit, day 1 visit and treatment weeks 1, 2, 4, 8 (and week 12 for Arm B patients and patients with compensated cirrhosis) and post treatment weeks 2, 4, 8, 12, and 24. The primary efficacy endpoint for GT1 HCV-infected patients without cirrhosis was to demonstrate non-inferiority of 8 weeks of G/P compared to 12 weeks of OBV/PTV/r in achieving SVR12 among patients in the intent-to-treat (ITT) population, defined as those who received at least one dose of study drug, excluding those with the NS5A Y93H baseline polymorphism (ITT–PS). A secondary endpoint is the percentage of all patients receiving 8 weeks of G/P achieving SVR12 including those with the Y93H polymorphism (ITT population). For GT1-infected patients with compensated cirrhosis, the primary efficacy endpoint was the percentage of patients achieving SVR12 in the ITT population. The secondary endpoints for all cohorts in this study were the percentage of patients with virologic failure during treatment and virologic relapse post treatment. Next-generation sequencing was conducted on HCV NS3 and NS5A genes from samples collected from all patients at baseline, and presence of HCV baseline polymorphisms was evaluated using a 15% detection threshold.

Blood samples for pharmacokinetic assessment of the study drugs were collected from patients during each study visit (G/P: *N* = 129 for patients without cirrhosis and *N* = 38 for patients with compensated cirrhosis; OBV/PTV/r: *N* = 52 patients without cirrhosis). Patients consenting to intensive pharmacokinetic sampling had samples drawn at the study day 1 (at hour 2, 4, and 6 h post-dose) and at week 4 visit at hour 0 (before study drug administration) and 2 and 4 h post-dose. Plasma concentrations for GLE, PIB, and OBV/PTV/r were summarized as steady-state trough levels (C_trough_) based on binning of pharmacokinetic samples using time of sample collection after dosing, such that concentrations that fall in the interval of 22–26 h after dosing were considered as C_trough_ for once daily dosing. Plasma concentrations were determined using a validated liquid chromatography assay.

Adverse events and laboratory tests were assessed and relationship to study drugs were determined by the clinical investigator throughout the treatment period and 24 weeks post-treatment for safety evaluations. Treatment emergent adverse events (TEAEs)/serious-TEAEs were collected from the time of study drug administration until 30 days following discontinuation of study treatment. After 30 days following completion of study treatment and throughout the post-treatment period, only SAEs were collected. All adverse events were graded according to Common Terminology Criteria for Adverse Events (CTCAE), version 4.0.

### Statistical analyses

The primary analysis was conducted after all enrolled patients had completed the post-treatment week 12 visit or prematurely discontinued from the study. Efficacy, safety, and demographic analyses were performed on the ITT population. For Substudy 1, the primary efficacy endpoint was conducted on the ITT–PS population. The difference in SVR12 rate between the two arms (Arm A minus Arm B) of Substudy 1 was computed as well as the two-sided 95% confidence interval using the normal approximation to the binomial distribution. Non-inferiority of Arm A versus Arm B was considered established if the lower bound of this confidence interval was above −10%. Efficacy was also assessed in the modified ITT (mITT) population, which excluded patients who did not achieve SVR12 due to reasons other than virologic failure. Safety analyses compared the rate of adverse events and laboratory abnormalities between treatment groups (Arm A vs. Arm B) with the use of Fisher’s exact test.

## Results

### Baseline patient demographics and viral characteristics

A total of 181 DAA-naïve patients with GT1 HCV infection without cirrhosis were enrolled and treated in Substudy 1; 129 in Arm A and 52 in Arm B. Of those, 64% and 73% were female and 27% and 29% were treatment-experienced with IFN/pegIFN ± RBV in Arms A and B, respectively. As is typical in the Japanese HCV population, most patients were elderly with a median age of 64 and 67 in the two arms, respectively. The mean HCV RNA at baseline was 6.1 ± 0.8 log_10_ and 6.2 ± 0.6 log_10_ IU/ml for the two arms, respectively. The NS5A Y93H polymorphism was present at baseline in 23 (18%) patients all of whom were assigned to Arm A. Four patients (3%) had GT1a infection (all in Arm A); the remaining patients in all arms had GT1b.

A total of 38 DAA-naïve patients with GT1 HCV infection and compensated cirrhosis were enrolled in Substudy 2. Of those, 55% were female and 32% were treatment-experienced. Mean HCV RNA at baseline was 6.0 ± 0.8 log_10_ IU/ml and median age was 73 years. The NS5A Y93H polymorphism was present at baseline in 16% of patients. Detailed patient demographic and baseline characteristics are outlined in Table 2.

**Table 2:** Baseline demographics and disease characteristics of GT1 HCV-infected patients without cirrhosis enrolled in Arms A and B, and GT1 HCV-infected patients with compensated cirrhosis enrolled in Arm C of CERTAIN-1

Characteristic	Substudy 1		Substudy 2
	Without cirrhosis		Compensated cirrhosis
	Arm A	Arm B	Arm C
	G/P	OBV/PTV/r	G/P
	8 weeks	12 weeks	12 weeks
	*N* = 129	*N* = 52	*N* = 38
Female, *n* (%)	82 (64)	38 (73)	21 (55)
Age, median (range), years	64 (21–86)	67 (31–81)	73 (48–85)
Age distribution, *n* (%)			
≥ 65 to < 75 years	37 (29)	23 (44)	15 (40)
≥ 75 years	26 (20)	7 (14)	16 (42)
BMI, mean ± SD, kg/m^2^	24 ± 4	23 ± 4	24 ± 5
IL28B non-CC genotype, *n* (%)	50 (39)	20 (39)	7 (18)
Treatment-naïve, *n* (%)	94 (73)	37 (71)	26 (68)
Treatment-experienced (IFN-based ± RBV), *n* (%)	35 (27)	15 (29)	12 (32)
HCV subtype, *n* (%)			
1a	4 (3)	0	0
1b	125 (97)	52 (100)	38 (100)
NS5A Y93H present^a^, *n* (%)	23 (18)	0	6 (16)
HCV RNA, mean ± SD, log_10_ IU/ml	6.1 ± 0.8	6.2 ± 0.6	6.0 ± 0.8
PPI use, *n* (%)	13 (10)	5 (10)	6 (16)
Liver protectant use, *n* (%)	39 (30)	15 (29)	23 (61)
Calcium channel blockers use, *n* (%)	25 (19)	4 (8)	14 (37)

*BMI* body mass index, *G/P* glecaprevir/pibrentasvir, *OBV/PTV/r* ombitasvir/paritaprevir/ritonavir, *IL28B* interleukin 28B, *PPI* proton pump inhibitor

^a^ Presence of Y93H in Substudy 1 determined by SRL using population sequencing, which has an approximate detection threshold of 15%. Presence of Y93H in Substudy 2 determined by next generation sequencing at 15% detection threshold

### Efficacy outcomes

**Fig. 2:**
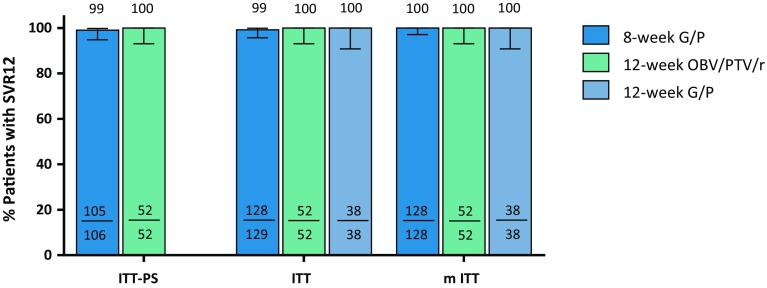
SVR12 rates for each arm in the ITT-PS, ITT and mITT populations. The error bars represent the 95% confidence intervals based on Wilson’s score method.  Arm A: 8-week G/P treatment; Arm B: 12-week OBV/PTV/r treatment; Arm C: 12-week G/P treatment. *SVR* sustained virologic response; *ITT* intent-to-treat; *ITT-PS* ITT population excluding patients with the NS5A Y93H baseline polymorphism; *mITT* ITT excluding patients who did not achieve SVR12 for reasons other than virologic failure

The CERTAIN-1 study met the primary endpoint, confirming that G/P for 8 weeks was non-inferior to OBV/PTV/r for 12 weeks, as the lower bound of the 95% confidence interval (CI) for the difference in SVR12 between Arms A and B (−0.9%; 95% CI −2.8%, 0.9%) was above the predefined threshold of −10%. 99.1% (105/106) of patients without cirrhosis in the ITT–PS population randomized to receive 8 weeks of G/P in Arm A achieved SVR12; no virologic failures occurred resulting in an SVR12 rate of 100% in the mITT population. The single patient not achieving SVR12 was lost to follow-up after achieving SVR4. One-hundred percent of patients with the Y93H baseline polymorphism (enrolled in Arm A) achieved SVR12, resulting in an overall SVR12 rate of 99.2% in Arm A, and 100% (52/52) of patients enrolled in Arm B and treated with OBV/PTV/r for 12 weeks achieved SVR12. All 38 (100%) HCV GT1-infected patients with compensated cirrhosis treated with G/P for 12 weeks achieved SVR12 (Fig. 2).

### Impact of baseline polymorphisms

Among GT1-infected DAA-naïve patients treated with G/P, Y93H in NS5A was detected in 16% (6/38) and 19% (23/124) in those with and without cirrhosis, respectively (Supporting Table 1). There were no virologic failures among GT1-infected DAA-naïve patients treated with G/P, therefore baseline polymorphisms, including Y93H, did not have an impact on treatment outcome. Y93H was not detected at baseline in any DAA-naïve patients treated with OBV/PTV/r, as per the enrollment criteria.

### Pharmacokinetic results

Following administration of G/P, GLE and PIB plasma concentrations attained steady state by the week 1 visit and remained constant throughout the treatment period (week 1–8 for DAA-naïve GT1 patients without cirrhosis and week 1–12 for DAA-naïve GT1 patients with compensated cirrhosis). No apparent accumulation was observed for GLE and PIB plasma concentrations in this study. GLE plasma concentrations were higher in patients with cirrhosis compared to patients without cirrhosis while PIB plasma concentrations were comparable between patients with and without compensated cirrhosis.

### Safety outcomes

TEAEs were experienced by 57% and 67% of patients in Arms A and B, respectively; 23% and 27% patients experienced at least 1 event that was considered study drug related, respectively; 66% of patients with compensated cirrhosis in Substudy 2 experienced TEAEs; 18% experienced at least one event that was considered study drug related (Table 3). No serious TEAEs occurred in patients (with or without cirrhosis) treated with G/P. Three patients (6%) receiving OBV/PTV/r (Arm B) had serious TEAEs that were assessed as not study-drug related in two patients and study-drug related in the third patient leading to treatment discontinuation on day 59; the patient went on to achieve SVR12. The smaller number of serious AEs observed in Arm A relative to Arm B was statistically significant (*p* = 0.023). One patient with compensated cirrhosis discontinued treatment at day 29 due to a grade 2 non-serious TEAE (drug eruption) that was assessed as study–drug related; this patient went on to achieve SVR12. The only TEAE occurring with a frequency > 10% in either arm of noncirrhotic patients was nasopharyngitis which was reported by 16% patients treated with G/P (Arm A) and 14% of patients treated with OBV/PTV/r (Arm B); malaise was reported by 11% of cirrhotic patients (Arm C). One TEAE (pyrexia) was statistically significantly different between Arm A and Arm B (0 vs. 6%, *p* = 0.023).

**Table 3:** Treatment-emergent adverse events

Event	Substudy 1		Substudy 2
	Without cirrhosis		Compensated cirrhosis
	Arm A	Arm B	Arm C
	G/P	OBV/PTV/r	G/P
	8 weeks	12 weeks	12 weeks
	*N* = 129	*N* = 52	*N* = 38
	*n* (%)	*n* (%)	*n* (%)
Any AE	74 (57)	35 (67)	25 (66)
Any drug-related AE	30 (23)	14 (27)	7 (18)
Any serious AE	0	3 (6)	0
Any DAA-related serious AE	0	1 (2)	0
Any AE leading to D/C of study drug	0	1 (2)	1 (3)^a^
Any AE leading to interruption of study drug	0	1 (2)	0
Common AEs (occurring in ≥ 5% and ≥ 2 patients in any arm)			
Nasopharyngitis	20 (16)	7 (14)	3 (8)
Malaise	3 (2)	0	4 (11)
Pruritus	8 (6)	5 (10)	2 (5)
Headache	6 (5)	5 (10)	1 (3)
Hypertension	4 (3)	4 (8)	1 (3)
Blood bilirubin increased	3 (2)	3 (6)	1 (3)
Cystitis	1 (1)	3 (6)	0
Pyrexia^†^	0	3 (6)	1 (3)
Cough	1 (1)	1 (2)	2 (5)
Rash	3 (2)	3 (6)	2 (5)
Atrial fibrillation	0	0	2 (5)
Head discomfort	0	0	2 (5)
Oropharyngeal pain	0	0	2 (5)
Pruritus generalized	0	0	2 (5)

*AE* adverse event, *DAA* direct-acting antiviral, *D/C* discontinuation

^†^ Difference between Arm A and Arm B was statistically significant (*p* = 0.023)

^a^ Discontinued treatment at day 29 due to a non-serious grade 2 AE (drug eruption) that was assessed as study–drug related by the treating physician, the patient achieved SVR12

For patients (with or without compensated cirrhosis) treated with G/P, no grade ≥ 3 abnormalities occurred in hemoglobin, alanine aminotransferase (ALT), aspartate aminotransferase (AST), or total bilirubin levels. For patients treated with OBV/PTV/r, one grade 3 increase in ALT level was reported. Table 4 shows the number and percentage of patients with laboratory abnormalities.

**Table 4:** Key post-baseline laboratory abnormalities

Laboratory abnormalities	Substudy 1		Substudy 2
	Without cirrhosis		Compensated cirrhosis
	Arm A	Arm B	Arm C
	G/P	OBV/PTV/r	G/P
	8 weeks	12 weeks	12 weeks
	*N* = 129	*N* = 52	*N* = 38
	*n* (%)	*n* (%)	*n* (%)
Hemoglobin			
Grade 2 (8–10 g/dl)	2 (1.6)	4 (7.7)	1 (2.6)
Grade ≥ 3 (< 8 g/dl)	0	0	0
Alanine aminotransferase			
Grade 2 (> 3–5 × ULN)	1 (0.8)	1 (1.9)	0
Grade ≥ 3 (> 5 × ULN)	0	1 (1.9)	0
Aspartate aminotransferase			
Grade 2 (> 3–5 × ULN)	1 (0.8)	1 (1.9)	0
Grade ≥ 3 (> 5 × ULN)	0	0	0
Total bilirubin			
Grade 2 (> 1.5–3 × ULN)	2 (1.6)	3 (5.8)	3 (7.9)
Grade ≥ 3 (> 3 × ULN)	0	0	0

*ULN* upper limit of the normal range

## Discussion

The CERTAIN-1 study met the primary endpoint in the ITT–PS population confirming non-inferiority of the 8-week treatment with G/P to the 12-week treatment with OBV/PTV/r in HCV GT1-infected patients without cirrhosis and without the NS5a Y93H polymorphism; 99.1% (105/106) of patients in the ITT–PS population achieved SVR12 and no virologic failures occurred. Previous studies have shown that lower SVR12 rates were achieved in patients with GT1b treated with OBV/PTV/r who have the NS5A Y93H polymorphism at baseline compared to HCV GT1b patients without this polymorphism [17]. As a result, JSH guidelines for the management of hepatitis C virus infection does not recommend treatment with OBV/PTV/r for patients with the NS5A Y93 polymorphism [1, 13]. Patients with Y93H in this study were assigned to treatment with G/P and 100% achieved SVR12, indicating the high potency of G/P against a hepatitis C virus containing the Y93H polymorphism, whose incidence rate in Japanese HCV patients is approximately 19% [18]. One-hundred percent of patients with compensated cirrhosis enrolled in Substudy 2 and treated with G/P for 12 weeks achieved SVR12. The high SVR12 rate observed in the overall population was also observed in other patient subgroups that have been previously considered difficult to treat, such as those with a high baseline viral load (i.e., ≥ 6 million IU/ml; *n* = 12), those with non-CC IL28B genotype (*n* = 57), those with prior IFN-based treatment experience (*n* = 48). These results indicate that G/P may allow for a simplified treatment algorithm without the need for NS5A Y93H polymorphism genotyping.

G/P was well tolerated in patients with and without compensated cirrhosis with no serious TEAEs reported, no grade ≥ 3 laboratory abnormalities in hemoglobin, bilirubin, ALT or AST levels, and one patient (< 1%) discontinuing study drug due to an TEAE. Only two TEAEs occurred with a frequency ≥ 10%; nasopharyngitis in non-cirrhotic subjects treated with G/P or OBV/PTV/r and malaise in cirrhotic subjects. Additionally, no patient experienced laboratory abnormalities indicating liver disease progression, no patient experienced hepatic decompensation, and no cases occurred that were consistent with drug-induced liver injury. The favorable tolerability profile, even in patients with compensated cirrhosis, suggests that treatment demands in patients who receive G/P may be reduced.

The reduced treatment duration of 8 weeks for GT1-infected patients without cirrhosis compared to recommended first-line treatment options for Japanese patients of 12 weeks of SOF/LDV, OBV/PTV/r, GZR+EBR, or DCV/ASV/BCV, is a significant reduction in treatment duration that in addition to convenience can help reduce the burden of managing concomitant medications, particularly in Japan’s HCV patient population where about 70% are older than 60 years [15]. In this study, 56% of patients treated with G/P were older than 65 years of age. Currently, no HCV treatment is approved in Japan with a duration of < 12 weeks.

Limitations of this study include the open-label design and the lack of an active comparator for patients with compensated cirrhosis. The use of objective laboratory-based efficacy endpoints and laboratory assessments for safety, however, mitigate these limitations. Despite the favorable results observed in this study, the relatively small number of patients in some subgroups (patients with a viral load ≥ 6 million log_10_ IU/ml and patients with GT1a HCV infection) precludes drawing further conclusions. However, high SVR12 rates have been observed in those subpopulations with a relatively large number of patients in studies outside Japan [19].

In summary, a 99.1% SVR12 rate was achieved in treatment-naïve and IFN ± RBV treatment-experienced (DAA-naïve) HCV GT1-infected patients without cirrhosis and without the Y93H NS5A baseline polymorphism, and 100% in those with the Y93H polymorphism following 8 weeks of treatment with G/P, with no virologic failures. Non-inferiority compared with 12 weeks of treatment with OBV/PTV/r was achieved. A 100% SVR12 rate was achieved in patients with compensated cirrhosis following 12 weeks of treatment with G/P. The regimen was well tolerated, with no serious AEs and low rates of liver-related laboratory abnormalities. G/P may therefore provide a compelling treatment option for this patient population with high efficacy and favorable tolerability, largely independent of baseline patient demographics and disease characteristics (including the NS5A Y93H baseline polymorphism), and a shorter treatment duration in patients without compensated cirrhosis than other available treatment options currently available in Japan.

## Electronic supplementary material

Below is the link to the electronic supplementary material.

Supplementary material 1 (DOCX 68 kb)
