# Supplementary material for: Efficacy and safety of glecaprevir/pibrentasvir in Japanese patients with chronic genotype 1 hepatitis C virus infection with and without cirrhosis
Source: J Gastroenterol. 2017 Sep 25;53(4):557–65. doi: 10.1007/s00535-017-1391-5 (PMC5866824; doi:10.1007/s00535-017-1391-5)
Supplement: Supplementary file 1 — Supplementary material 1 (DOCX 68 kb) [file 535_2017_1391_MOESM1_ESM.docx]

# Online Supporting Information

# Efficacy and Safety of Glecaprevir/Pibrentasvir in Japanese Patients with Chronic Genotype 1 Hepatitis C Virus Infection with and without Cirrhosis

Chayama, Kazuaki^1^; Suzuki, Fumitaka^2^; Karino, Yoshiyasu^3^; Kawakami, Yoshiiku^1^; Sato, Ken^4^; Atarashi, Tomofumi^5^; Naganuma, Atsushi^6^; Watanabe, Tsunamasa^7^; Eguchi, Yuichiro^8^; Yoshiji, Hitoshi^9^; Seike, Masataka^10^; Takei, Yoshiyuki^11^; Kato, Koji^12^; Alves, Katia;^12^ Burroughs, Margaret^12^, Redman, Rebecca^12^; Pugatch, David L^12^; Pilot-Matias, Tami J^12^; Krishnan, Preethi^12^;  Oberoi, Rajneet K^12^; Xie, Wangang^12^; Kumada, Hiromitsu^2^

## Table of contents

[Eligibility Criteria 2](#_Toc479596269)

[Inclusion 2](#_Toc479596270)

[Exclusion 4](#_Toc479596271)

[Supporting Figure 1. Patient disposition. 6](#_Toc479596272)

[Supporting Table 1. Prevalence of Baseline Polymorphisms. 7](#_Toc479596273)

## Eligibility Criteria

### Inclusion

1. Japanese male or female subjects at least 18 years of age at time of screening.

2. Female who is not of childbearing potential or of childbearing potential and sexually active with male partner(s) and currently using at least one effective method of birth control at the time of screening and agrees to practice one effective method of birth control while receiving study drugs starting with Screening and for 30 days after stopping study drug.

3. Sexually active males must be surgically sterile, or if sexually active with female partner(s) of childbearing potential must agree to practice one effective form of birth control starting with Screening and through 30 days after completion of the study drug.

4. Screening central laboratory result indicating HCV single genotype infection for the appropriate treatment arm, without co-infection of any other genotype.

5. Subject has positive anti-HCV Ab and plasma HCV RNA viral load ≥ 1000 IU/mL at Screening Visit.

6. Chronic HCV infection defined as one of the following:

● Positive for anti-HCV antibody (Ab) and/or HCV RNA at least 6 months before Screening.

● A liver biopsy consistent with chronic HCV infection.

7. Subject must be:

● HCV DAA treatment-naïve (i.e., patient has not received a single dose of any approved or investigational DAA). Prior HCV treatment using IFNs with or without ribavirin, is acceptable. Previous HCV IFN based treatment must have been completed ≥ 2 months prior to screening.

8. Must voluntarily sign and date an informed consent form, approved by an Institutional Review Board (IRB)/Independent Ethics Committee (IEC) prior to the initiation of any screening or study specific procedures.

9. Subjects must be able to understand and adhere to the study visit schedule and all other protocol requirements.

In addition to Inclusion Criteria 1 through 9, subjects without cirrhosis must meet the following criteria:

10. Subject must be documented as non-cirrhotic, defined as meeting one of the following criteria:

● A liver biopsy within 24 months prior to or during screening demonstrating the absence of cirrhosis, e.g., a METAVIR, Batts-Ludwig, Knodell, IASL, Scheuer, New Inuyama or Laennec fibrosis score of ≤ 3, Ishak fibrosis score of ≤ 4;

● A FibroScan® score of < 12.5 kPa within 6 months of Screening or during the Screening Period;

● A screening FibroTest score of ≤ 0.72 and Aspartate Aminotransferase to Platelet Ratio Index (APRI) ≤ 2;

● A screening Discriminant Score (z) less than zero, according to the following formula: z = 0.124 × [gamma-globulin (%)] + 0.001 × [hyaluronate (μg × l^–1^)] –0.075 × [platelet (× 10^4^ cells/mm^3^)] – 0.413 × gender (male, 1; female, 2) – 2.005.

In addition to Inclusion Criteria 1 through 9, subjects with compensated cirrhosis must meet the following criteria:

11. Subject must be documented as cirrhotic, defined as meeting one of the following criteria:

● A liver biopsy within 24 months prior to or during screening demonstrating the presence of cirrhosis, e.g., a METAVIR, Batts-Ludwig, Knodell, IASL, Scheuer, New Inuyama fibrosis score > 3 (including 3 – 4 or 3/4), or Laennec fibrosis score of > 3, Ishak fibrosis score of > 4;

● A FibroScan® score of ≥ 14.6 kPa within 6 months of Screening or during the Screening Period;

● A screening FibroTest score of ≥ 0.73 and Aspartate Aminotransferase to Platelet Ratio Index (APRI) > 2;

● A screening Discriminant Score (z) greater than zero, according to the following formula: z = 0.124 × [gamma-globulin (%)] + 0.001 × [hyaluronate (μg × l^–1^)] –0.075 × [platelet (× 10^4^ cells/mm^3^)] – 0.413 × gender (male, 1; female, 2) – 2.005.

12. Absence of hepatocellular carcinoma (HCC) as indicated by an ultrasound, computed tomography (CT) scan or magnetic resonance imaging (MRI) showing no evidence of HCC within 3 months prior to Screening or an ultrasound with no evidence of HCC at Screening. Subjects who have an ultrasound with results suspicious of HCC followed by a subsequent CT or MRI with no evidence of HCC will be eligible for the study.

### Exclusion

1. Female who is pregnant, planning to become pregnant during the study, or breastfeeding; or male whose partner is pregnant or planning to become pregnant during the study.

2. Recent (within 6 months prior to study drug administration) history of drug or alcohol abuse that could preclude adherence to the protocol in the opinion of the investigator.

3. Positive test result at Screening for hepatitis B surface antigen (HBsAg) or anti human immunodeficiency virus antibody (HIV Ab).

4. Requirement for and inability to safely discontinue contraindicated medications or supplements at least 2 weeks or 10 half-lives (whichever is longer) prior to the first dose of any study drug.

5. Clinically significant abnormalities, other than HCV-infection, based upon the results of a medical history, physical examination, vital signs, laboratory profile, and a 12-lead electrocardiogram (ECG) that make the subject an unsuitable candidate for this study in the opinion of the investigator, including, but not limited to:

● Uncontrolled diabetes as defined by a glycated hemoglobin (hemoglobin A1C) level > 8.5% at the Screening Visit.

● Active or suspected malignancy or history of malignancy (other than basal cell skin cancer or cervical carcinoma in situ) in the past 5 years, or any history of HCC.

● Uncontrolled cardiac, respiratory, gastrointestinal, hematologic, neurologic, psychiatric, or other medical disease or disorder, which is unrelated to the existing HCV infection.

6. Any cause of liver disease other than chronic HCV-infection, including but not limited to the following:

● Hemochromatosis, alpha-1 antitrypsin deficiency, Wilson's disease, autoimmune hepatitis, alcoholic liver disease, or steatohepatitis considered to be the primary cause of the liver disease rather than concomitant/incidental with HCV infection.

7. History of solid organ transplantation.

8. Receipt of any investigational product within a time period equal to 10 half-lives of the product, if known, or a minimum of 6 weeks (whichever is longer) prior to study drug administration.

9. Consideration by the investigator, for any reason, that the subject is an unsuitable candidate to receive G/P.

10. History of severe, life-threatening or other significant sensitivity to any study drugs or their excipients.

11. Patients who can't participate in study per local law.

12. Any current or past clinical evidence of Child-Pugh B or C classification or clinical history of decompensated liver disease such as ascites noted on physical exam, hepatic encephalopathy or variceal bleeding.

13. Screening laboratory analyses showing any of the following abnormal laboratory results:

● Estimated glomerular filtration rate (eGFRJ): < 30 mL/min/1.73 m^2^

● Albumin: < LLN for non-cirrhotics, < 2.8 g/dL for cirrhotics

● International normalized ratio (INR): ≥ 1.2 for non-cirrhotics, ≥ 1.8 for cirrhotics (Subjects with a known inherited blood disorder and INR ≥ 1.2 may be enrolled with permission of the AbbVie TA MD.)

● Hemoglobin: < 10 g/dL

● Platelets: < 90,000 cells per mm^3^ for non-cirrhotics, < 50,000 cells per mm^3^ for cirrhotics

## **Supporting Figure 1.** Patient disposition.

327 patients screened

32 patients failed screening:

24 Failed incl./excl. criteria

8 Withdrew consent

129 patients completed treatment

129 patients assessed for efficacy and safety

38 patients with GT1 and compensated cirrhosis

(Arm C)

38 patients assigned to treatment with G/P

(Arm C)

38 patients completed treatment

38 patients assessed for efficacy and safety

182 patients with GT1 w/o cirrhosis enrolled in Arms A and B

129 patients assigned to treatment with G/P

(Arm A)

52 patients completed treatment

52 patients assessed for efficacy and safety

53 patients assigned to treatment with OBV/PTV (Arm B)

75 patients of other subgroups analyzed elsewhere

## Supporting Table 1. Prevalence of Baseline Polymorphisms.

| **Table 3. Prevalence of Baseline Polymorphisms^a^** | | | |  |
| --- | --- | --- | --- | --- |
| **Target** | **Polymorphism** | **Arm A**  **G/P GT1 DAA-naïve non-cirrhotic, n (%)**  **N=125^b^** | **Arm B**  **OBV/PTV/r GT1 DAA-naïve non-cirrhotic, n (%)**  **N=49** | **Arm C**  **G/P GT1 DAA-naïve cirrhotic, n (%)**  **N=38** |
| NS3 | D168E | 1 (0.8) | 1 (2.0) | 1 (2.6) |
| NS5A | L31M | 4 (3.2) | 1 (2.0) | 2 (5.3) |
|  | Y93H | 21 (16.9) | 0 (0) | 6 (15.8) |

^a^Specific polymorphisms relative to reference sequence using a 15% detection threshold at the following amino acid positions:  155, 156, 168 in NS3; 31, 93 in NS5A.

**^b^**N=124 for NS5A
